# Case report: breast cancer associated with contralateral tuberculosis of axillary lymph nodes

**DOI:** 10.1186/1477-7819-11-43

**Published:** 2013-02-25

**Authors:** Muna M Baslaim, Shefaa A Al-Amoudi, Masoud A Al-Ghamdi, Abdullah S Ashour, Taha S Al-Numani

**Affiliations:** 1 King Fahd General Hospital, P. O. Box: 51652, Jeddah 21553, Saudi Arabia

**Keywords:** Breast cancer, Tuberculosis, Axillary lymph nodes

## Abstract

**Background:**

Breast cancer coexisting with tuberculous axillary lymph nodes is rare.

**Case report:**

We report a 69 years old Yemeni patient with a left breast invasive ductal carcinoma associated with contralateral tuberculous axillary lymph nodes containing microcalcifications mimicking malignancy. The patient had to be investigated for the possibility of bilateral breast cancer since she had no history of previous exposure to tuberculosis.

**Conclusion:**

Tuberculosis involving lymph nodes can create a diagnostic dilemma in the presence of a malignant process. The presence of calcifications in lymph nodes should raise the possibility of tuberculosis even in the absence of contact history with tuberculosis.

## Background

The synchronous occurrence of carcinoma and tuberculosis is unusual. The earliest known report of such a case is that of Pilliet and Piatot in 1897 [1]. After that and in 1899, Warthin reported the first case of coexisting tuberculosis and cancer in axillary lymph nodes [2]. Tuberculosis can produce masses and nodes that can imitate or complicate staging of the neoplastic disease [3].

We report a case of breast cancer with contralateral tuberculous axillary lymph nodes that created a diagnostic dilemma and raised the possibility of a bilateral breast cancer.

## Case presentation

A 69-year-old Yemeni woman presented to the breast clinic with a left breast painless mass increasing gradually in size over a few months. Lately she had noticed that the left nipple started to ulcerate and retract. Eight years ago, she had had colonic carcinoma, which was managed with left hemi-colectomy followed by chemotherapy and radiotherapy. She was from a high socioeconomic class, living in Saudi Arabia for more than 20 years and did not recall contact with patients with tuberculosis. Examination of the left breast showed a retracted ulcerated nipple; the skin was thickened making with the underlying mass an irregular large palpable area measuring 15 × 12 cm in maximum dimensions and there were no palpable axillary lymph nodes. Right breast examination was unremarkable but there was a rounded firm mobile 2-cm lymph node high up in the right axilla.

**Figure 1:**
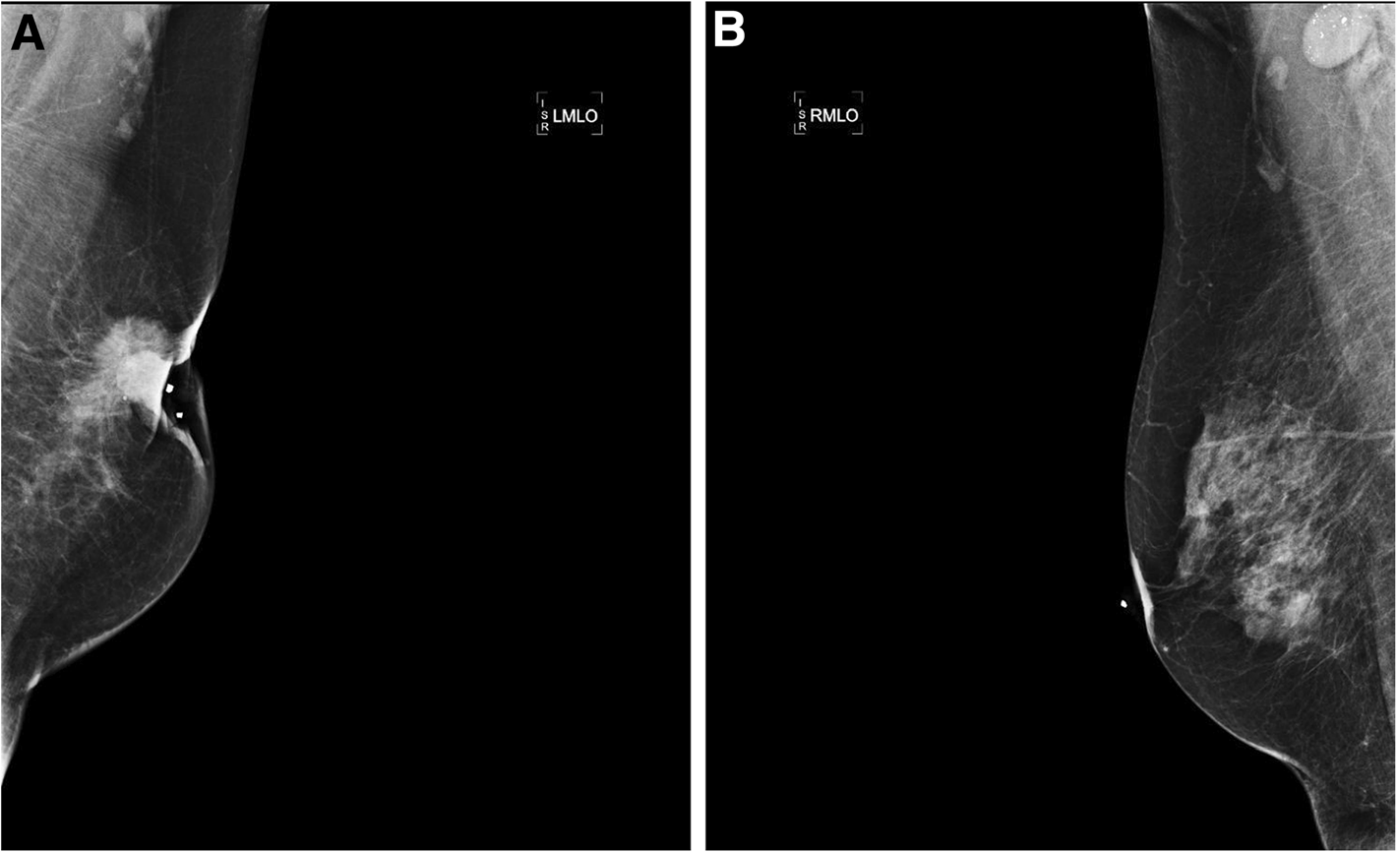
**Bilateral mammography A. Left-sided mammogram showing a stellate lesion in the retro-areolar area with skin involvement and nipple retraction. B**. Right-sided mammogram showing a large rounded axillary lymph node with calcifications.

Bilateral mammogram showed a left breast stellate retro-areolar lesion with nipple retraction (Figure 1A). The right breast was normal, however, there was a suspicious large rounded dense right axillary lymph node containing multiple calcifications (Figure 1B). Bilateral breast magnetic resonance imaging (MRI) showed multiple enhanced large suspicious right axillary lymph nodes; the largest one measured 2.3 cm with no evidence of a primary right breast lesion. Computed tomography (CT) showed multiple enlarged right axillary lymph nodes with calcifications. CT of the chest, abdomen and pelvis, as well as a bone scan showed no evidence of other pathology or distant metastases.

**Figure 2:**
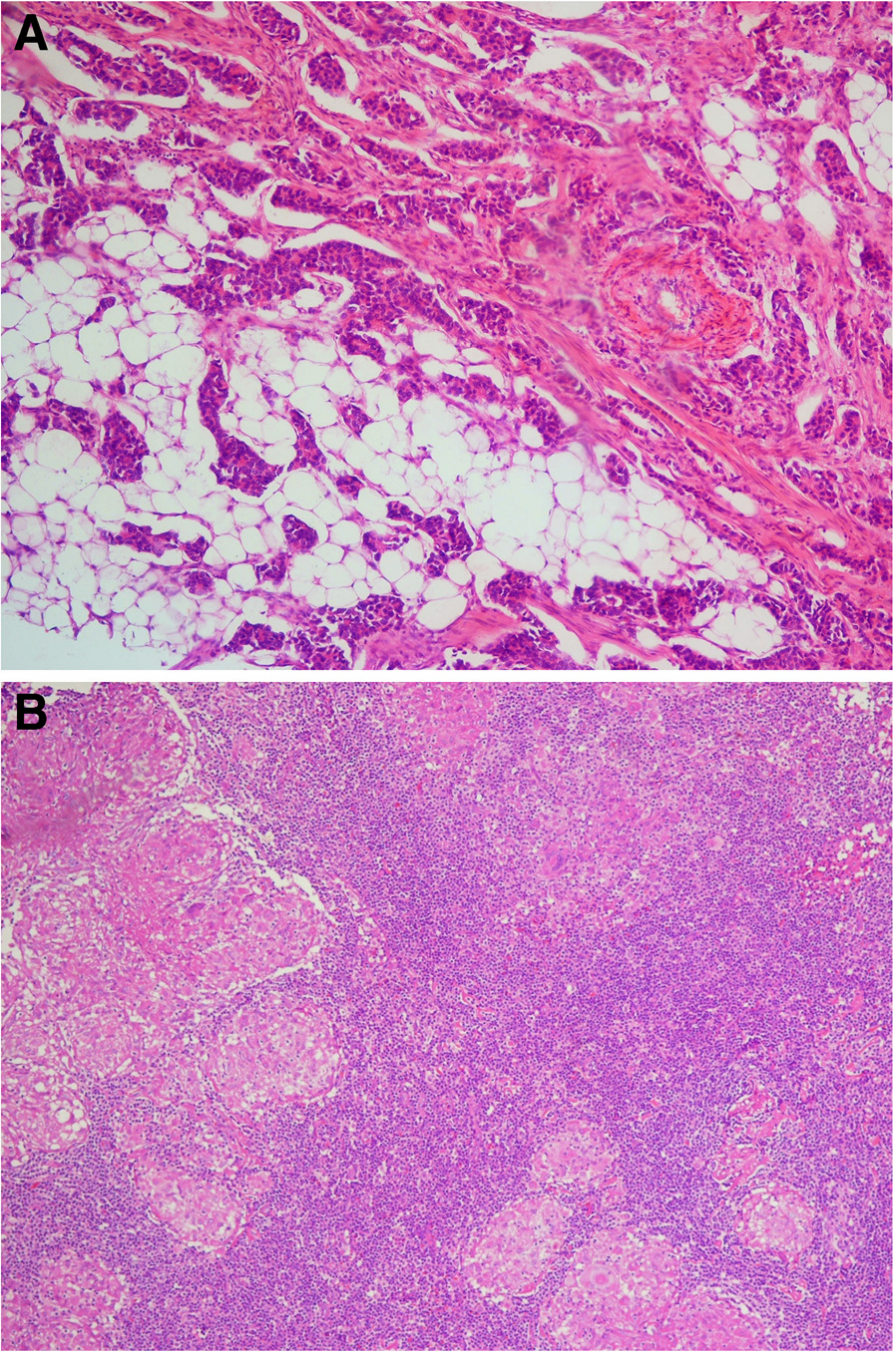
**Histopathology of the breast carcinoma and lymph nodes A. Invasive ductal carcinoma of the left breast infiltrating surrounding fat and stroma (haematoxylin and eosin stain, 10× magnification). B**. Lymph node containing multiple well-defined granulomas with central caseation containing giant cell (haematoxylin and eosin stain, 10× magnification).

Tru-cut needle biopsy of the left breast mass showed invasive ductal carcinoma (IDC), grade II, and fine needle aspiration (FNA) of the right axillary lymph node was insufficient for diagnosis. Repeat FNA was not attempted. The patient was reluctant to receive neo-adjuvant chemotherapy since she had a prior history of chemotherapy as a treatment for colonic cancer. She underwent left modified radical mastectomy and right axillary lymph node excision. Final histopathology showed left breast IDC, grade II (Figure 2A), with metastases in 10 lymph nodes out of 16 dissected. The lesion in the right axilla was a group of matted lymph nodes forming one mass; there were granulomatous lesions with caseation consistent with tuberculosis (Figure 2B).

The coexistence of tuberculosis and malignancy has been reported in the literature mainly with Hodgkin's lymphoma, sarcoma, leukemia, or lung cancer. It is least prevalent in patients with carcinoma of the colon, bladder, uterus, breast, prostate, and kidney [3].

Most of the reported cases of breast cancer with concomitant tuberculous axillary lymph nodes were of ipsilateral involvement [4-9]. Our patient created a diagnostic dilemma clinically and radiologically, since a malignant right axillary lymph node was suspected with no evidence of a primary lesion in the breast; hence a bilateral breast cancer was suspected. Moreover, breast MRI did not exclude malignant right axillary lymph nodes. Yang *et al*. reported a case of tuberculous axillary lymph node that was misinterpreted by ^18^F- fluorodeoxyglucose positron emission tomography (FDG-PET) as a malignant metastatic disease from a possibly occult breast cancer [10].

Extensive surgery such as axillary dissection can be avoided by clinically suspecting granulomatous disease with assessment of intraoperative frozen sections.

## Conclusion

In cancer patients, a tuberculous lymph node should be suspected whenever an enlarged lymph node shows calcifications on radiography, even without history of exposure to tuberculosis.

## Consent

Written informed consent was obtained from the patient for publication of this report and any accompanying images.

## Abbreviations

CT: Computed tomography;FDG-PET: ^18^F-fluorodeoxyglucose positron emission tomography;FNA: Fine needle aspiration;IDC: Invasive ductal carcinoma;MRI: Magnetic resonance imaging

## Competing interests

The authors declare that they have no competing interests.

## Authors’ contributions

MB, SA, MA, TA, and AA were involved in the conception of the report and acquisition of data. SA, MA, and TA were involved in the acquisition of images. MB, and AA drafted the manuscript. MB, SA, MA, TA, and AA corrected and revised the manuscript. MB, SA, MA, TA, and AA approved the final version for publication. All authors read and approved the final manuscript.
